# Convergence of emerging technologies: Development of a risk‐based paradigm for marine mammal monitoring for offshore wind energy operations

**DOI:** 10.1002/ieam.4532

**Published:** 2021-11-15

**Authors:** A. Michael Macrander, Louis Brzuzy, Kaustubha Raghukumar, Damian Preziosi, Craig Jones

**Affiliations:** ^1^ Integral Consulting Inc. Seattle Washington USA; ^2^ Shell Exploration and Production Company Houston Alaska USA; ^3^ Integral Consulting Inc. Santa Cruz California USA; ^4^ Integral Consulting Inc. Berlin Maryland USA

**Keywords:** Marine mammals, Mitigation, Monitoring, Risk assessment, Wind energy

## Abstract

The ability to gather real‐time and near real‐time data on marine mammal distribution, movement, and habitat use has advanced significantly over the past two decades. These advances have outpaced their adoption into a meaningful, risk‐based assessment framework so critically needed to support society's growing demands for a transition to increased reliance on renewable energy. Marine acoustics have the capacity to detect, identify, and locate vocalizations over broad areas. Photogrammetric and image processing increases the ability to visually detect animals from surface or aerial platforms. Ecological models based on long‐term observational data coupled with static and remotely sensed oceanographic data are able to predict daily and seasonal habitat suitability. Extensive monitoring around anthropogenic activities, combined with controlled experiments of exposure parameters (i.e., sound), supports better informed decisions on reducing effects. Population models and potential consequence modeling provide the ability to estimate the significance of individual and population exposure. The collective capacities of these emerging technical approaches support a risk ranking and risk management approach to monitoring and mitigating effects on marine mammals related to development activities. The monitoring paradigm related to many offshore energy‐related activities, however, has long been spatially limited, situationally myopic, and operationally uncertain. A case evaluation process is used to define and demonstrate the changing paradigm of effective monitoring aimed at protecting living resources and concurrently providing increased certainty that essential activities can proceed efficiently. Recent advances in both technologies and operational approaches are examined to delineate a risk‐based paradigm, driven by a diversity of regional data inputs, that is capable of meeting the imperative for timely development of offshore wind energy. *Integr Environ Assess Manag* 2022;18:939–949. © 2021 The Authors. *Integrated Environmental Assessment and Management* published by Wiley Periodicals LLC on behalf of Society of Environmental Toxicology & Chemistry (SETAC).

## Key Points

- This paper makes the case that the national policy and mandate for development of offshore wind resources can be facilitated by the updating of marine mammal monitoring approaches through the incorporation of emerging technologies and a regional risk‐based approach.
- There are currently a number of existing and developing efforts to collect information on marine mammals in the Atlantic Outer Continental Shelf that can be updated and incorporate into a regional monitoring effort through collaboration and integration.
- The regulatory construct is adaptable to a regional risk‐based approach, the implementation of which would require acceptance by regulators and stakeholders and relief for project proponents.

## INTRODUCTION

The outer continental shelf of the United States has been assessed to be a world‐class wind energy resource on both the Atlantic and Pacific coasts (Bureau of Ocean Energy Management, 2019a). Ongoing public demand for clean energy alternatives, coupled with increased technological advancements, has greatly expanded the potential for US offshore wind. The US Department of Energy estimates a technical potential of 2058 gigawatts (GW) of offshore wind resource capacity accessible in US coastal waters using existing technology (US Department of Energy, 2016). This resource represents a potential energy output that is nearly double the total electric generation in the United States in 2015 and is located close to 80% of existing power consumption. By June 2019, the Bureau of Ocean Energy Management (BOEM) reported the existence of 15 commercial leases in the Atlantic Outer Continental Shelf (AOCS) that can provide 21 GW of generating capacity (Bureau of Ocean Energy Management, 2019a). Most recently (as of August 2021), BOEM reports 21 commercial leases and three wind energy call areas for the AOCS (Bureau of Ocean Energy Management, 2021). Momentum toward rapid development of offshore wind energy has been given a clear mandate by the current US Administration through Executive Order 14008 (Executive Office of the President, 2021a) and an announcement of actions and policies through a Fact Sheet (Executive Office of the President, 2021b) that establish the goal of deploying 30 GW of offshore wind energy by 2030.

The coastal waters that are targeted to receive much of this development, including the AOCS, are also important use areas that support fisheries (commercial and recreational), vessel traffic, and military mission needs critical to the well‐being and prosperity of the nation (Bureau of Ocean Energy Management, 2019a). In addition to supporting some of the nation's most valuable fisheries and fishing communities, the coastal and marine environment of the AOCS supports a rich ecosystem of global significance, including such iconic species as the North Atlantic right whale (*Eubalaena glacialis*), Atlantic salmon (*Salmo salar*), Atlantic sturgeon (*Acipenser oxyrinchus oxyrinchus*), and Atlantic cod (*Gadus morhua*), and five species of sea turtles including the green (*Chelonia mydas*), hawksbill (*Eretmochelys imbricata*), loggerhead (*Caretta caretta*), leatherback (*Dermochelys coriacea*), and Kemp's ridley (*Lepidochelys kempii*) sea turtles (National Oceanic and Atmospheric Administration, 2020a).

In addition to the North Atlantic right whale, Hayes et al. (2019) lists 38 individual marine mammal taxa as occurring in the waters of New England and the Mid‐Atlantic. Seven species stocks, including blue whale (*Balaenoptera musculus*), fin whale (*Balaenoptera physalus*), North Atlantic right whale, sei whale (*Balaenoptera borealis*), sperm whale (*Physeter macrocephalus*), false killer whale (*Pseudorca crassidens*), and common bottlenose dolphin (*Tursiops truncatus*), meet one or more of the criteria for listing of “Strategic” under the Marine Mammal Protection Act (MMPA). Five species (blue whale, fin whale, North Atlantic right whale, sei whale, and sperm whale) are listed as endangered under the Endangered Species Act. Of these marine mammals, four listed species (fin whale, North Atlantic right whale, sei whale, and sperm whale) were identified by the National Marine Fisheries Service (NMFS) through an Endangered Species Act Section 7 Consultation as being potentially subject to effects related to wind generation development (National Marine Fisheries Service, 2013).

The MMPA as amended in 2018 (U.S.C. 16 1361 et seq., 2018) established a moratorium on the taking of marine mammals, with “taking” defined broadly to include fatality, injury, and harassment or disturbance. Although activities related to the development and operation of offshore wind energy facilities present relatively low risk of fatality or injury of marine mammals, there is potential for disturbance including displacement or disruption of important behavior patterns. The MMPA also has a provision that allows commercial activities to request the authorization of incidental harassment of small numbers of individuals in cases in which it can be ascertained that the result of the activity is likely to have a negligible impact on species or stocks. When issuing incidental harassment authorizations (IHAs), regulatory agencies, such as NMFS, typically work with applicants to define appropriate monitoring and mitigation measures to be implemented in conjunction with the activity to provide protection to the resources and affirmative reporting on compliance. The MMPA also states that marine mammals should be protected and populations “encouraged to develop to the greatest extent feasible commensurate with sound policies of resource management” and that affirmative actions may be developed to enhance populations and ecosystem health.

A nexus exists between responsible resource management and authorization of activities that have been identified as a national priority with an aggressive timeline. Within that nexus may lie largely unexplored opportunities to incorporate emerging monitoring capacities and risk assessment coupled with programmatic risk management.

This article is organized as follows: Existing standards for marine mammal monitoring and mitigation are reviewed, followed by an overview of current marine mammal surveys and monitoring efforts in the AOCS region. Current and emerging technologies for marine mammal monitoring are then reviewed, followed by recommendations for case studies that argue for regional, risk‐based paradigms for monitoring marine mammal populations.

## EXISTING STANDARD FOR MARINE MAMMAL MONITORING AND MITIGATION

Monitoring and mitigation practices designed to reduce the potential effects of industrial development, transportation, and military operations have developed, and become relatively standard practice, over the past 50 years (ACCOBAMS Scientific Committee, 2017; Bröker, 2020; Castellote, 2007; International Association of Oil and Gas Producers, 2017; Joint Nature Conservation Committee, 2010a, 2010b, 2010c, 2017; Martin et al., 2014; Nowacek & Southall, 2016; Nowacek et al., 2013; Weir & Dolman, 2007). Table 1 provides a brief list and descriptions of the most common monitoring and mitigation measures recommended by project operators and regulatory entities. The use of protected species observers (PSOs; also referred to as marine mammal observers) has been a common form of observational monitoring, coupled with operational mitigations, since the 1980s. Passive acoustic monitoring (PAM) has been a more common addition to PSOs since the mid‐2000s in an effort to provide monitoring capacity during periods of limited visibility. The number, type, and extent of these measures within a specific project are often the result of an evaluation of the proposed activity's intensity, spatial area, and duration, as well as the likelihood of occurrence of marine mammal populations within the project area. Larger, more intensive projects occurring within the range of large numbers or highly vulnerable marine mammals may require more extensive monitoring and mitigation based on an assessment of relative risk. Often, the extent of monitoring and mitigation measures may also be a result of engagement and negotiation with regulators and stakeholders with the goal of allowing a project to go forward while lowering overall and cumulative risk (Chou et al., 2021).

**TABLE 1:** Standard marine mammal mitigation and monitoring measures employed for offshore development

Monitoring and mitigation type	Description
**Programmatic**	
Activity and equipment design	Evaluation of the activity(ies) to be conducted and equipment required to accomplish tasks identifying opportunities to reduce or minimize disturbance potential
Estimation of effects area	Use of models and other means to estimate propagation of sound and other disturbance metrics around activities for the evaluation of potential effects and establishment of safety or exclusion zones
Sound baffling	Use of measures (e.g., bubble curtains) around certain activities (e.g., pile driving) to reduce the transmission of sound energy within water and reduce propagation and potential for effects
Spatial restrictions	Limitation of operations within specific areas, for example, habitats, feeding areas, breeding or natal areas; often coupled with temporal restrictions
Temporal restrictions	Limitations of operations during specific perceived sensitive periods (e.g., breeding, natal, feeding, migration); often coupled with spatial restrictions
**Observation**	
Qualified human observers	Observers on duty during operational periods to observe, collect data, and (potentially) call for operational mitigations
Passive acoustic monitoring (PAM)	Use of arrays of acoustic hydrophones and software to detect, identify, and (potentially) localize marine mammals close to operations
Aerial monitoring with human observers	Aerial overflights performed at relatively low altitude to detect and observe marine mammals close to operations and (potentially) call for mitigations
**Operational**	
Speed limits	Limitation of vessel speed (usually to no more than 10 knots) when operating within established sensitive areas, as identified through spatial or temporal criteria
Safety or exclusion zones	Delineation of areas (distances) around planned activities within which exposure to generated sound energy may produce deleterious effects on marine mammals. Such exposures are avoided through implementation of mitigation measures
Pre‐activity monitoring	Monitoring of safety or exclusion zones and general proximity of the operational area for a period prior to initiation of an activity to determine whether marine mammals are present and potentially susceptible to effects
Activity delay	Delay of initiation of activity to allow marine mammals observed within potential‐effects areas to leave the area
Ramp up	Gradual initiation of operations beginning with least intensive and progressing to most intensive to allow marine mammals present to detect and move away. Sometimes referred to as soft start
Shutdown	Cessation of activities as the result of observation (either visual or acoustic) of marine mammals within, approaching, or close to a safety or exclusion zone

To date, survey‐related monitoring projects authorized and implemented in association with wind energy development in the AOCS have been focused on marine site characterization, primarily through the use of relatively low‐energy survey equipment. A review of the IHAs issued by NMFS indicates that monitoring observation includes PSOs in all cases, PAM in approximately one‐third of the projects, and no aerial monitoring. The operational mitigation measures listed in Table 1 are required in all of the IHAs. It is more difficult to assess whether programmatic mitigation measures (e.g., survey timing, constraint of source energy) are in place, as many of these are incorporated in the planning stages of project development and application processes and may not be captured within the IHAs.

Although the implementation of established monitoring and mitigation measures has allowed wind energy and other industrial activities to go forward, while effectively protecting the marine mammal resources, they are not without their shortcomings. These shortcomings are particularly evident when there is a priority for efficacious implementation of a national energy policy while operating within the range of a critically endangered species, the North Atlantic right whale.

Even with the inclusion of PAM in addition to PSOs, monitoring around industry activities is typically limited spatially to gathering information effectively to within a few hundred meters to kilometers (Smith et al., 2020) and, temporally, to brief observation encounters. Even these limited distances can be challenged by conditions that restrict visibility (e.g., darkness, fog, or precipitation) or the background acoustic environment (Verfuss et al., 2018). The implementation of mitigation measures is not usually informed by what may be larger patterns of distribution, movement, behavior, or ecological parameters as might be derived from more regional‐based monitoring such as the Atlantic Marine Program for Protected Species (AMAPPS; National Marine Fisheries Service, 2020a), the Northwest Large Whale Pelagic Survey and Collaborative Aerial and Acoustic Surveys for Large Whales and Sea Turtles (Kraus et al., 2016), and the US Northeast Passive Acoustic Sensing Network (NEPAN; Van Parijs et al., 2015). Although all IHAs require a report that provides an account of observations and mitigation measures implemented, these data have not, to date, been utilized in any meaningful manner to improve the understanding of marine mammal abundance, distribution, behavior, or ecology or to improve overall management or conservation as directed by guidance for monitoring programs (National Marine Fisheries Service, 2021a). At the same time, staffing and equipping two to four PSOs and PAM operators on multiple vessels is costly and produces, in some cases, hundreds of operational delays and temporary shutdowns resulting in expanded project timelines, uncertainty, and costs. Elliott et al. (2019) point out that these measures are implemented with little actually known about their efficacy in protecting marine vertebrates. They also suggest the use of technological and programmatic advances to improve monitoring and mitigation. Fleishman et al. (2016) discuss the need for monitoring approaches that are able to assess population‐level response to human activity. Chou et al. (2021) point out that research is needed to understand the trade‐off between noise‐effect reduction over more extended duration versus less reduction over shorter periods.

Nowacek et al. (2016) suggest the use of technological advances to improve the study of behavior and produce conservation advances. As the nation moves toward an aggressive timeline for implementation of renewable wind energy, which potentially brings with it a concomitant benefit to conservation of marine mammal populations by limiting the extent of climate‐related habitat effects, it may be opportune to examine alternatives to standard practices for application of monitoring and mitigation around offshore renewable energy development.

## EXISTING MARINE MAMMAL SURVEY AND MONITORING IN THE AOCS

The public, academic, and private sectors are all actively engaged in investigating and monitoring the marine mammal populations of the AOCS. Of necessity mandated by the responsibility to advise conservation and management decision‐making, public and private sector methods and technologies deployed are expected to be less experimental or leading edge than academic‐based advancement. All three sectors are, however, contributing meaningfully to the development and utilization of new investigatory, monitoring, and mitigation approaches in this rapidly changing field.

Since 2010, NMFS has collaborated with BOEM and the Department of the Navy to conduct the AMAPPS, a program that utilizes both aerial and vessel‐based observations (National Marine Fisheries Service, 2020a). In addition to collecting data related to abundance and distribution of marine mammals and other protected species, the AMAPPS also captures and develops a photogrammetric database of marine mammals to generate population and health metrics. The collaborative North Atlantic right whale monitoring effort also relies intimately on AMAPPS and other associated aerial and vessel‐based observation programs to provide data related to the investigation of this critically endangered species (Oleson et al., 2020).

The National Oceanic and Atmospheric Administration (NOAA) and associated parties have invested in the acquisition and utilization of acoustic data within the AOCS. The NOAA has developed and implemented the NEPAN, composed of numerous acoustic recorders to provide archived and near‐real‐time data from the Gulf of Maine to the New York Bight (Van Parijs et al., 2015).

The Northeast Large Whale Pelagic Survey Collaborative Aerial and Acoustic Surveys for Large Whales and Sea Turtles (Kraus et al., 2016) have combined aerial survey data collection with acoustics data collection to provide a comprehensive baseline characterization of the abundance, distribution, and spatiotemporal occurrence of marine mammals, with a focus on large endangered whales and sea turtles, in the Massachusetts and Rhode Island wind energy areas and surrounding waters. Both BOEM and the New York State Energy Research and Development Authority have conducted aerial surveys in areas of potential wind energy development (Normandeau Associates, 2012; Normandeau Associates & APEM, 2018).

In collaboration with NOAA, BOEM, and conservation organizations, the North Atlantic Right Whale Catalog has been established and curated at the New England Aquarium in Boston. This catalog contains more than 73 000 images of more than 720 individuals (some of which are known to be deceased) dating back to 1935. While the annual aerial surveys are being conducted, NMFS also opportunistically acquires images of whales with a specific emphasis on North Atlantic right whales. The NMFS also conducts specific flights and vessel cruises to established hot spots of North Atlantic right whale concentration for the purpose of acquiring images to be added to the catalog. Images from the catalog have been utilized for a number of analytic purposes relevant to the assessment, management, and conservation of North Atlantic right whale stocks including high confidence population estimates and trends utilizing photo‐based mark and recapture methods (Pace et al., 2017), identification of important habitat (Pace & Merrick, 2008), and health assessments (Pettis et al., 2004).

Roberts et al. (2016) combined the results of 35 years of visual line‐transect surveys with fine‐scale environmental covariates to produce density models for the US Atlantic and Gulf of Mexico, establishing a basis for model‐based evaluation of the potential effects of environmental change on marine mammal populations. This modeling effort converts line‐transect data to probabilistic projections of density and distribution across the AOCS that are based on the combination of visual observations and oceanographic parameters.

Under Renewable Energy Program Regulations (30 CFR 585), wind energy developers are required to conduct site characterization surveys to evaluate the impact of proposed activities on physical, biological, and socioeconomic resources. These characterizations employ vessel, aerial, and acoustic survey methods to develop datasets that are relevant to the specific area of planned operation as well as the AOCS in general. In addition to these surveys, there is an increasing trend for offshore wind operators to enter into data‐sharing agreements (Executive Office of the President, 2021b), collaborative research agreements (Vineyard Wind, 2019), and investigative partnerships with research institutions (Rutgers Center for Ocean Observing Leadership, 2021) that promise to utilize expanding ranges of technology and methods to provide data on the status, distribution, and behavior of marine mammals in the AOCS.

## EMERGING TECHNOLOGIES FOR MARINE MAMMAL MONITORING AND MITIGATION

Although the body of science and ongoing studies related to the monitoring, management, and conservation of marine mammals of the AOCS is quite extensive and well established, changing environmental and operating conditions encourage the continuing adaptation of these programs to improve data‐collection methods, reduce costs, and reduce investigator safety risk profiles. The incorporation of and transition to emerging methods and technologies in ecological monitoring are consistent with NOAA's stated science enterprise goals (National Oceanic and Atmospheric Administration, 2020b). It has also been specifically called for by a number of science review panels including the NMFS Expert Working Group for the North Atlantic Right Whale (Oleson et al., 2020). It is important to review emerging and advanced technologies similar to Nowacek et al. (2016) that can modify, complement, or replace existing investigative and monitoring strategies in a manner that improves information on distribution and ecology of marine mammals in the AOCS. Many of these technologies are already broad used within the marine science community and are ready for operational incorporation and deployment. Others are on the verge of greatly expanded utility through innovation.

By definition, emerging technologies go well beyond the customary approach to monitoring and mitigation, both changing the ways in which some data are collected and, in some cases, expanding to a more inclusive collection and incorporation of ecological, management, and risk assessment methods consistent with Nowacek and Southall (2016) and Chou et al. (2021). It is through the integration of these more inclusive data types in a real‐time, risk‐based paradigm that would most successfully meet the dual goals of protecting marine mammal resources and achieving timely development of wind energy.

### Unmanned systems

Autonomous systems are aerial or underwater vehicles that are able to operate without the presence of a person on board to provide steering and operational support. The use of unmanned, or unoccupied, systems for the collection of ecological and marine science data has been expanding rapidly. At this point, the diversity of platforms, sensors, and capabilities is nearly as broad as the field of marine data acquisition with an ever‐increasing availability of unmanned solutions to collect physical and biological oceanographic data (Bellingham, 2009; Johnson, 2019). Recent policy statements and guidance by NOAA underscore the importance of the continued development and transition to unmanned systems to the future delivery of that agency's mission (National Oceanic and Atmospheric Administration, 2020c).

#### Unmanned aerial systems

Unmanned aerial systems come in a wide range of sizes and configurations including rotary‐winged and fixed‐winged (Johnson, 2019). They vary in capacity for onboard payloads, range and speed of flight, and ability to operate independently. They have been used successfully to accomplish a diversity of tasks including collection of tissue and respiration samples (Pirotta et al., 2017), photogrammetry for health assessments (Christiansen et al., 2020), and, to a lesser extent, in conducting monitoring and population assessments (Bröker et al., 2019; Koski et al., 2009, 2013).

#### Autonomous underwater vehicles

Similar to unmanned aerial systems, the autonomous underwater vehicles include a broad spectrum of vehicles with the common aspect being that these vehicles are able to traverse areas of coastal and open‐ocean waters in both submerged and surface modes, while carrying a potential variety of data‐collection payloads. Autonomous underwater vehicles are often used as survey platforms to map the seabed or characterize physical, chemical, or biological properties of the water. Sidescan sonar payloads accommodate seabed mapping. Acoustic Doppler profilers help to characterize ocean currents. Conductivity‐temperature‐depth instruments provide insights into salinity patterns, temperature, and depth across marine areas and habitats. Dissolved oxygen, nutrient, and chemical constituent data can be sampled and associated with spatiotemporal patterns. Sonar instruments, such as multibeam sonar, can collect data on fishery and plankton distributions. The PAM payloads can detect, record, and potentially identify and localize marine mammal calls and other forms of marine bioacoustics (Bellingham, 2009). At this point, there are few forms of marine monitoring data that cannot be collected directly or inferred from the payload systems of autonomous underwater vehicles.

### Image capture and analysis

The employment of human observers (PSOs) for the collection of marine mammal data from land, vessel, and aerial platforms has been the standard for as long as such surveys and monitoring have been conducted. There are, however, a number of drawbacks to the use of human observers. There is variable expertise and ability among PSOs. Attention and focus may vary through time. Frequent duty rotations require multiple PSOs on a project to maintain high levels of effectiveness. Observations occur over limited periods and generate no capacity for review or verification. The PSOs may be exposed to harsh conditions and, in the case of aerial observations, high levels of risk.

Digital image capture and analysis have been advancing quickly over the past decades and are being incorporated into marine mammal studies and monitoring. Multiple investigators have explored the possibility of replacing PSOs on aircraft (Ferguson et al., 2018; Hodgson et al., 2013; Koski et al., 2013; Sweeney et al., 2016), and Normandeau Associates has used it extensively in collecting data in support of wind energy development in the AOCS. Camera systems have also been developed to assist in land‐ and vessel‐based observation of marine mammals (Weissenberger et al., 2011; Zitterbart et al., 2013). These systems largely utilize infrared imagery to detect and, with image processing, identify marine mammals. Blows of large cetaceans that differ significantly from the surrounding ambient temperatures are detectable with this method, as are portions of bodies above the sea surface.

### PAM

Passive acoustic monitoring moorings and towed arrays are used both to monitor specific areas within the AOCS and to enhance the success of vessel‐based, line‐transect surveys. With the continued expansion of acoustics data, it is likely that improved system capability, strategic deployment, and analytic tools, and the utility of this information, are likely to continue to grow. As an example of this utility, PAM arrays have been demonstrated to be able to produce population estimates (Davis et al., 2017; Latusek‐Nabholz et al., 2020; Marques et al., 2009). Oleson et al. (2020) expressed the belief that well‐designed, long‐term coordinated visual and PAM efforts may yield benefits for identifying or predicting distribution shifts, supporting population analyses, and estimating demographic rates for the North Atlantic right whale population.

Increasing interest in the development of energy resources (particularly wind energy) within the AOCS is likely to significantly expand the use and presence of PAM efforts within the area. Passive acoustic monitoring is a standard part of recommended marine mammal monitoring programs (Bureau of Ocean Energy Management, 2019b) and has already been used extensively by private wind energy operators. A strategic approach to these various monitoring programs, in combination and consideration of existing efforts by public and stakeholder entities, can produce a powerful cumulative dataset.

## CASE STUDIES IN EFFECTIVE MONITORING AND RISK ASSESSMENT

The opening of regional areas of the marine continental shelf to development of renewable energy provides due cause for use of innovative approaches to regulatory compliance and effect mitigation. Three brief case studies described below demonstrate how changing paradigms in resource development and risk assessment and representation are improving marine mammal protection.

From 2005 until 2015, the oil and gas industry committed significant resources and effort to exploring for commercial oil and gas in the offshore Chukchi and Beaufort seas of Alaska. Some of these companies (Shell, ConocoPhillips, and Statoil) developed a collaborative research effort, the Chukchi Sea Environmental Studies Program (CSESP). The CSESP represented an alternative to the typical prospect‐specific site characterizations required by regulation and conducted by industry in that it spanned across and between multiple development areas and adopted an integrated ecosystem approach (Hopcroft & Day, 2013). The CSESP and other of the companies' research initiatives have led to nearly 100 peer‐reviewed publications and greatly increased the understanding of the physical and ecological factors that drive this ecosystem. During much of this period, state and federal governments were also funding research in the area (Dunton et al., 2014). Eventually, industry and government initiatives became more collaborative and co‐funded, resulting in the establishment of a data‐sharing agreement in which all of the industry‐produced data became available through an ocean observing portal. Shell also entered into a collaborative research agreement with the indigenous communities of the North Slope of Alaska (North Slope Borough, 2021).

Ecological risk assessment has been utilized extensively as a process for estimating the probabilities of undesired ecological effects resulting from physical, chemical, and biological stressors present in the environment (Suter & Norton, 2019). Southall et al. (2016, 2017) presented the work of an expert working group in which they utilized an ecological risk assessment approach involving species' population trends, life‐history parameters, and other relevant biological and environmental contexts as well as quantification of uncertainty. This report was utilized and cited by NMFS extensively in the development of incidental take regulations for the conduct of geophysical surveys in the Gulf of Mexico (National Marine Fisheries Service, 2021b). Other nations and jurisdictions are increasingly applying risk assessment and risk management based approaches to evaluate and manage offshore wind development (Heinis et al., 2019). Whale Safe is an innovative integration of near real‐time data streams from PAM, habitat suitability modeling, and visual observation to create a tool to help prevent ship strikes within the Santa Barbara Channel (University of California Santa Barbara, 2021). The risk‐based tool integrates data from near real‐time acoustics detections (Baumgartner et al., 2019) with visual observations generated by trusted observers and recorded within the Whale Alert (National Marine Fisheries Service, 2020b) and Spotter Pro (Conserve IO, 2020), and habitat model‐based estimates of probability (Abrahms et al., 2019) to generate ratings of likelihood of whale presence and maps of known and likely occurrence. In addition to generating ratings and maps of whale presence, Whale Safe also monitors vessel traffic within the Santa Barbara Channel and rates vessel operators based on compliance with NOAA‐identified voluntary vessel speed reduction zones. By integrating data on the presence and intensity of threat of vessel strike with data on probability of species' occurrence, the Whale Safe model represents a multispecies tool for assessing and managing risk in near real time over large areas of habitat and development.

## DISCUSSION

The development of offshore wind energy within the AOCS represents the convergence of two strategic national priorities—the development of renewable, noncarbon‐based energy resources and the management and recovery of critical marine mammal resources. Accomplishment of both goals within an aspirational timeline mandates the very real need for development and implementation of new approaches to protecting and recovering marine mammals while providing greater operational certainty for development activities.

With respect to the AOCS, there clearly exists a strong foundation of data, tools, and models upon which to build. For the AOCS, there exists a rich data trove on marine mammal presence, movement, and biology as corroborated by such programs as AMAPPS (National Marine Fisheries Service, 2020b), NEPAN (Van Parijs et al., 2015), the Northeast Large Whale Pelagic *S*urvey (Kraus et al., 2016), and the New York State Energy Research and Development Authority (Normandeau Associates & APEM, 2018). There are several other examples that add to the data. As briefly described in this article, a host of traditional tools (e.g., use of PSOs) and emerging technologies (e.g., PAM through near real‐time, broad‐area networks and autonomous platforms) used to detect and monitor marine mammals is also widely available and rapidly expanding the body of relevant datasets. Adding to these data and tools are habitat‐modeling approaches to predict spatiotemporal risk contours on both long‐term and near real‐time bases.

The extent of environmental data for the AOCS is expanding rapidly in conjunction with wind energy development. In addition to conducting the requisite environmental characterization to comply with the National Environmental Policy Act, energy development interests have established high standards for investigating the potential interaction between sensitive marine mammal resources and development projects. The Western Gray Whale Advisory Panel initiated by Sakhalin Energy and the International Union for the Conservation of Nature established a collaborative studies and operations advisory capacity to ensure the long‐term conservation of western gray whales (Martin‐Mehers, 2016). Since its establishment in 2004, the panel has worked with Sakhalin Energy not only to implement recommendations that allowed oil and gas activities to go forward while protecting the whales, but also to greatly expand the knowledge of this critically endangered population. Exploration efforts in the Chukchi and Beaufort seas between 2005 and 2015 fostered a rapid expansion of studies of those ecosystems with a strong focus on the marine mammal resources and their interaction with oil and gas activities. Given the increasing number of collaboration agreements already established by wind energy interests in the AOCS, it is evident that the current wealth of data and conservation planning will expand rapidly over the coming decade.

A recent state‐of‐the‐science workshop hosted by the New York State Energy Research and Development Authority utilized a highly collaborative effort among representatives of the research, regulatory, industry, and key stakeholder communities to develop and publish a series of examinations of existing science and scientific needs for marine resources (https://www.nyetwg.com/2020-workgroups) including a report dedicated to marine mammals (Southall et al., 2021). Among the principal findings of this workshop was an emphasis on coordination, data standardization, and transparency.

Although broadly implemented and generally accepted for application in offshore development activities, the monitoring paradigm related to many offshore, energy‐related activities has long been spatially and temporally limited, situationally myopic, and operationally uncertain. Observations are limited in scope in both space and time, and the range of mitigation options is narrow, most commonly consisting of cessation or delay of activities when marine mammals are detected close to operations. The incorporation of risk assessment and risk management in the procedures is extremely limited to nonexistent, resulting in an approach that is highly precautionary.

At the same time, the development of risk‐based monitoring and mitigation approaches related to marine mammals is expanding. Several examples, including Whale Safe (University of California Santa Barbara, 2021), Sanguinetti et al. (2021), and Sèbe et al. (2019), are well along toward establishing regional monitoring systems and risk‐based mitigation criteria for marine mammals. Most of these current efforts are related to managing the risk of vessel strikes. Numerous efforts are also underway to monitor marine mammal distribution, activities, and ecology over relatively large areas, many of which incorporate real‐time identification, processing, and notification (National Marine Sanctuaries, 2021).

The ocean is not uniform in terms of relative risk across space, time, species, species sensitivity, or human activity. Distribution of marine mammals within and among species varies in space and time, resulting in spatiotemporal variations in relative risk of exposure to stressors. Different species, populations, and species groups have different levels of sensitivity resulting from interspecific differences of physiology, behavior, and population status. Ecological variations in space and time create conditions more, or less, favorable to long‐term maintenance of healthy populations (Roberts et al., 2016). Consequences of exposure either at the individual or the population levels vary across activity patterns and life‐history stages (e.g., breeding, feeding, or migration; National Research Council, 2005). Likewise, industry activities vary in consequences of exposure in frequency intensity, duration, and spatial extent.

Protocols for the assessment of ecological risk normally follow a tiered approach through the evaluation of (1) the potential for exposure (pathway), (2) characterization of exposure, (3) effects of exposure, and (4) integration of exposure potential–exposure magnitude–effects consequence profiles (Suter & Norton, 2019). Through the collection of data and application of modeling, risk can be estimated and represented in the form of two‐ to four‐dimensional risk contours, including time, that allow decision‐makers to establish and address mitigation priorities. The risk profile of an area containing multiple species and subject to constantly changing ecological profiles and industrial development is extremely dynamic, requiring near real‐time data and projection capacities. Given the potential large‐scale movements of marine mammals, coupled with the broad spatial coverage of future offshore wind activities, a regional approach is needed. Of course, within the regional approach lie the implicit assumptions of data sharing and transparency for the benefit of all stakeholders.

Despite the inherent complexity of developing such a regional, real‐time risk evaluation and communication system, the initial development of such a system can be based on existing data streams, rapidly emerging technologies, and numerical modeling. The capacity to understand the distribution and activities of marine mammals is expanding rapidly through real‐time acoustics networks, visual and machine‐assisted observation (including vessel, aerial, and satellite), and monitoring of physical and biological ocean conditions in support of habitat modeling. The capacity to track and monitor human and development‐related activities through vessel tracking, acoustic monitoring, and acoustic modeling is well established. As wind energy development activities and monitoring efforts increase over the coming decade, these capacities are highly likely to improve significantly.

Although existing standards of marine mammal monitoring and mitigation around industrial activities are widely accepted and practiced throughout the world, they mostly represent a set of best available and precautionary practices that have been in use for decades. There are a few demonstrable cases in which populations of marine mammals, such as the western gray whale (Nowacek & Southall, 2016), have increased within areas where energy development has occurred concurrent with application of strict standards of monitoring and mitigation. It is also apparent that effects on marine mammal behaviors often diminish significantly within only a few kilometers of development activities (Ireland et al., 2017). Despite these examples, however, the efficacy of existing monitoring practices is largely inferred. Likewise, the real impacts of existing practices on cost and timeline of offshore development are also poorly understood.

Despite these challenges, existing standards for monitoring and mitigation have the benefit of being widely accepted, and criteria for implementation are relatively clear. In addition to the fact that the risk projection capacity is in its early days, implementation within a framework of regulatory compliance would require significant dialogue and consideration of the application of risk‐based monitoring to existing regulations. The National Marine Fisheries Service (2021a) provides guidance on what information needs to be included in an application for incidental take authorizations. The guidance is clear in stating that applicants should describe those measures that will be implemented to avoid and mitigate potential effects and the monitoring that will be conducted to (1) support the implementation of mitigations, (2) provide quantifiable data on marine mammal encounters and mitigations implemented, and (3) increase understanding of marine mammals and interactions with human activities. The guidance goes further to suggest that certain measures (e.g., use of biological observers, safety zones, shutdowns, and PAM) are relatively standard. The guidance does not, however, indicate that these standard practices are required, therefore accommodating the possibility that the requirements may be met through alternative adaptive measures. It is potentially through the development of an adaptive regional risk assessment and risk management program, which incorporates a broad spectrum of data from a number of monitoring platforms from multiple collaborators and innovative modeling, that practitioners and stakeholders can move beyond the limitations of existing standards.

The strength of a regional risk‐based system, though, would have the ability to “see” marine mammal behavior patterns on a large scale and in relation to human activities, thus having the capacity to improve in terms of both conserving the resources and accommodating the implementation of lower risk activities. Such a monitoring tool would further provide a legacy for broad conservation monitoring and evaluation of regional stress factors well beyond the development of wind energy. Such a risk‐based approach would also provide for differential application of mitigation measures based on situational conditions (e.g., species distribution, behavior, susceptibility, and conservation priority).

It will be critical to define the processes by which mitigation can be applied on a more regional scale, as opposed to within the confines of an ongoing development activity. There is an inherent reality that project developers would need to achieve real benefits in terms of implementation timelines and cost to transition from the existing, albeit uncertain and burdensome, measures to a process that more accurately applies the level of mitigation that is needed for specific situations.

Though it is not clear what entities would be ultimately responsible for establishing and administering a regional, risk‐based monitoring and mitigation system, significant momentum surrounds the development of wind energy in the AOCS to create new and increasing collaborations among interested parties. The unique environmental, economic, and social imperatives of wind energy may provide sufficient impetus for a risk‐based approach that is more finely attuned to ecological and development realities.

## DISCLOSURE STATEMENTS

Three of the authors work, either currently or in the recent past, for international energy companies, all of which are transitioning from oil and gas to renewable energy. All three have extensive experience developing and managing marine mammal monitoring and mitigation and studies programs, as well as working with multiple stakeholders, for example, regulators, researchers, and environmental NGOs. While working for energy companies, they represent the interests of all parties within the conservation network and are influenced most by their experience of what works. All of the authors have careers and expertise in ocean science and marine acoustics in particular and are actively engaged in working to advance science and achieve effective solutions.

## Data Availability

There were no data collected in the development of this work product.
